# Impact of heart rate on adverse events in patients with non-valvular atrial fibrillation: Subanalysis of the J-RHYTHM Registry

**DOI:** 10.1016/j.ijcha.2022.101148

**Published:** 2022-11-17

**Authors:** Eitaro Kodani, Hiroshi Inoue, Hirotsugu Atarashi, Ken Okumura, Takeshi Yamashita, Hideki Origasa, on behalf of the J-RHYTHM Registry Investigators

**Affiliations:** a Department of Cardiovascular Medicine, Nippon Medical School Tama Nagayama Hospital, Tokyo, Japan; b Saiseikai Toyama Hospital, Toyama, Japan; c AOI Hachioji Hospital, Tokyo, Japan; d Saiseikai Kumamoto Hospital, Kumamoto, Japan; e The Cardiovascular Institute, Tokyo, Japan; f The Institute of Statistical Mathematics, Tokyo, Japan

**Keywords:** Atrial fibrillation, Heart rate, Thromboembolism, Major hemorrhage, Death

## Abstract

**Background:**

Although heart rate (HR) is reportedly associated with major cardiovascular outcomes in the general population, its impact on adverse events in patients with non-valvular atrial fibrillation (NVAF) remains controversial. Thus, we performed *post hoc* analyses of data from the J-RHYTHM Registry to clarify this in patients with NVAF.

**Results:**

Of 7406 outpatients with NVAF from 158 institutions, 6886 (age, 69.8 ± 9.9 years; men, 70.8%), in whom both baseline HR and HR-end (at the time closest to an event or at the last visit of follow-up) were measured during the two-year follow-up period or until the occurrence of an event, constituted the study group. The baseline HR and HR-end values were 72.5 ± 13.3 bpm and 73.3 ± 13.3 bpm, respectively. Thromboembolism, major hemorrhage, all-cause death, and cardiovascular death occurred in 117 (1.7%), 130 (1.9%), 157 (2.3%), and 58 (0.8%) patients, respectively. Baseline HR was not associated with any adverse event, whereas HR-end (per 1-bpm increase) was significantly associated with an increased incidence of all adverse events. Furthermore, the highest quartile of HR-end (≥80 bpm) was independently associated with the incidence of major hemorrhage (adjusted odds ratio [OR], 2.90; 95% confidence interval [CI], 1.69–4.96; P < 0.001), all-cause death (OR, 3.42; 95% CI, 1.99–5.88; P < 0.001), and cardiovascular death (OR, 5.07; 95% CI, 1.49–17.22; P = 0.009) compared with the second quartile (64–71 bpm), even after adjusting for known confounding factors, HR-controlling drug use, and systolic blood pressure-end.

**Conclusions:**

In patients with NVAF, HR-end was significantly associated with adverse events independent of systolic blood pressure-end, whereas baseline HR was not.

## Highlights

• Baseline heart rate was not associated with any adverse event in non-valvular atrial fibrillation.
• A higher heart rate at the time closest to an event was associated with incidence of adverse events.
• A heart rate < 64 bpm at the time closest to an event was associated with cardiovascular death.
• These findings were almost consistent, irrespective of atrial fibrillation type.

## 1 Introduction

Atrial fibrillation (AF) is a common arrhythmia and major risk factor for ischemic stroke and heart failure [1], [2]. Since heart rate (HR) control can reduce patient symptoms, improve quality of life (QOL) and exercise tolerance, and ameliorate heart failure, HR control has been a front-line therapy in the management of AF. Thus, previous guidelines empirically recommended strict HR control [3]. However, this may result in bradycardia, syncope, and the occasional need for pacemaker implantation. In the Rate Control Efficacy in Permanent Atrial Fibrillation II (RACE II) trial [4], lenient HR control (resting HR < 110 beats per minute [bpm]) was not inferior to strict HR control (resting HR < 80 bpm and HR during moderate exercise < 110 bpm) in terms of cardiovascular morbidity and mortality. Therefore, lenient HR control has become an acceptable initial approach regardless of heart failure status in the 2020 European Society of Cardiology Guidelines [5]. Although HR is reportedly associated with cardiovascular mortality in the general population [6], its impact on adverse events in patients with non-valvular AF (NVAF) remains controversial. Therefore, here we performed *post hoc* analyses of data from the J-RHYTHM Registry [7] to clarify the effects of HR in patients with NVAF.

## 2 Methods

### 2.1 Study design of the J-RHYTHM Registry

The J-RHYTHM Registry was a nationwide prospective observational study that clarified the status of anticoagulation therapy and determined the optimal anticoagulation therapy in Japanese patients with AF [7]. The study design and baseline patient characteristics were reported elsewhere [7], [8]. The study protocol conformed to the Declaration of Helsinki and was approved by the ethics committee of each participating institution. Written informed consent was obtained from all participants at the time of enrollment. A consecutive series of outpatients with AF of any type were enrolled from 158 institutions between January and July 2009, regardless of their use of anticoagulants, antiarrhythmic drugs, and HR-controlling drugs. All drugs and their dosages were selected at the discretion of the treating physicians. Patients with valvular AF (mechanical heart valve and mitral stenosis) were excluded from the subanalysis. HR and blood pressure (BP) were measured in each patient at the time of enrollment (baseline) and at each follow-up visit. Patients with NVAF, in whom both baseline HR and HR-end (HR at the time closest to an event or at the last visit of follow-up) were measured during the two-year follow-up period or until the occurrence of an event, were included in this *post hoc* analysis,

### 2.2 Follow-up and definition of endpoints

Patients were followed up for two years or until an event occurred, whichever came first. The primary endpoints were as follows: thromboembolism including symptomatic ischemic stroke, transient ischemic attack (TIA), and systemic embolic events; major hemorrhage including intracranial, gastrointestinal, and other hemorrhages requiring hospitalization; and all-cause and cardiovascular deaths. The diagnostic criteria for each event were described elsewhere [7], [8].

### 2.3 Patient groups

Patients were categorized by quartiles of baseline HR (<63, 63–70, 71–79, and ≥ 80 bpm) or HR-end (<64, 64–71, 72–79, and ≥ 80 bpm). In addition, based on the target HR values in the RACE II trial [4] and clinical relevance, the patients were arbitrarily divided into 4 baseline HR groups (<60, 60–79, 80–109, and ≥ 110 bpm). Quartiles or groups of baseline HR and HR-end were labeled the lowest, second, third, and highest, respectively, in ascending order from the lowest to the highest. For the subgroup analyses, patients were further divided into the paroxysmal AF and non-paroxysmal (persistent and permanent) AF subgroups based on baseline AF type.

### 2.4 Statistical analysis

Data are presented as mean ± standard deviation (SD) or number (percentage). To compare patient characteristics and two-year event rates among the quartiles or four groups, a trend analysis was performed using the Cochran-Armitage test for categorical variables or the Jonckheere-Terpstra test for continuous variables, as appropriate. A Cox proportional hazards model was used to investigate the influence of baseline HR on adverse events. The Cox proportional hazards assumption was verified using log–log survival curves in the study outcomes. Univariable and multivariable logistic regression analyses were performed to determine the influence of the HR-end on adverse events. The risk of baseline HR or HR-end for adverse events in each quartile or group was expressed as hazard ratios or odds ratios (ORs) and 95% confidence intervals (CIs) versus the second quartile. Explanatory variables for multivariable analysis were adopted from well-known risk factors used in our previous subanalyses for hypertension and BP [9], [10]. These included components of the CHA_2_DS_2_-VASc score (congestive heart failure, hypertension, age ≥ 75 years, diabetes mellitus, history of stroke or TIA, vascular disease [coronary artery disease], age 65–74 years, female sex) [11], warfarin and antiplatelet use, AF type, and systolic BP-end (BP at the time closest to an event or at the last visit of follow-up) (Model 1). An additional model included the variables of Model 1 plus hypertrophic cardiomyopathy (HCM), chronic obstructive pulmonary disease (COPD), calcium channel blocker (CCB) use, creatinine clearance (CrCl), body mass index (BMI), and hemoglobin levels as explanatory variables (Model 2) based on baseline patient characteristics and the results of our previous subanalyses [12], [13], [14]. Since β-blockers and digitalis have a potential effect on HR, the use of these drugs was also included in Model 2. The same analyses were performed using the continuous values of the baseline HR and HR-end as a sensitivity analysis. In addition, all analyses were performed separately for patients with paroxysmal and non-paroxysmal AF. Two-tailed P-values of < 0.05 were considered statistically significant. All statistical analyses were performed using SPSS software (version 23.0; IBM Corporation, Armonk, NY, USA).

## 3 Results

Of the 7937 patients with AF enrolled in the J-RHYTHM Registry, 421 (5.3%) with valvular AF were excluded, and 110 (1.5%) were lost to follow-up. Of the remaining 7406 patients with NVAF, 520 (7.0%) for whom fewer than two HR measurements were taken during the follow-up period were excluded. Consequently, 6886 patients (age, 69.8 ± 9.9 years; men, 70.8%) were included in this subanalysis.

### 3.1 Patient characteristics and medications

The clinical characteristics and medications of the 6886 patients are listed in Supplementary Table 1. HR was measured 14.4 ± 5.2 times during the follow-up period. Median interval between the day of an event and the day of HR-end measurement was 0 day (interquartile range, −4 and 0 days). Baseline HR and HR-end values were 72.5 ± 13.3 bpm and 73.3 ± 13.3 bpm, respectively (Supplementary Table 1).

The patient characteristics and medications in baseline HR quartiles are shown in Table 1. The prevalence of non-paroxysmal (persistent and permanent) AF, COPD, heart failure, and diabetes mellitus showed significant increasing trends across the quartiles, resulting in higher CHADS_2_ scores for patients in the higher quartiles. In contrast, the prevalence of coronary artery disease and HCM showed decreasing trends across quartiles (Table 1). Among the antiarrhythmic and HR-controlling drugs, CCB use was significantly more frequent among patients in the higher HR-end quartiles (Table 1). The patient characteristics and medications in HR-end quartiles are shown in Supplementary Table 2. As for the baseline HR quartiles in Table 1, many variables showed significant trends across the HR-end quartiles (Supplementary Table 2).

**Table 1:** Patient characteristics and medications in baseline heart rate quartiles.

	**Lowest** **(<63 bpm)**	**Second** **(63–70 bpm)**	**Third** **(71–79 bpm)**	**Highest** **(≥80 bpm)**	***P*-value for trend**
Number of patients	1621	1739	1631	1895	
Age, years	69.5 ± 9.4	70.2 ± 10.1	69.7 ± 10.1	69.7 ± 10.1	0.697
Sex, male	1174 (72.4)	1213 (69.8)	1158 (71.0)	1329 (70.1)	0.262
Body mass index, kg/m^2^ (n = 5979)	23.6 ± 3.6	23.6 ± 3.6	23.6 ± 3.6	23.7 ± 4.9	0.507
Type of atrial fibrillation					
Paroxysmal	934 (57.6)	770 (44.3)	501 (30.7)	448 (23.6)	0.046
Persistent	197 (12.2)	210 (12.1)	221 (13.5)	384 (20.3)	
Permanent	490 (30.2)	759 (43.6)	909 (55.7)	1063 (56.1)	
Comorbidities					
Coronary artery disease	179 (11.0)	208 (12.0)	163 (10.0)	174 (9.4)	0.018
Cardiomyopathy	133 (8.2)	156 (9.0)	138 (8.5)	146 (7.7)	0.457
HCM	70 (4.3)	63 (3.6)	56 (3.4)	44 (2.3)	0.001
DCM	63 (3.9)	93 (5.3)	82 (5.0)	102 (5.4)	0.081
Congenital heart disease	21 (1.3)	26 (1.5)	16 (1.0)	27 (1.4)	0.950
COPD	21 (1.3)	25 (1.4)	32 (2.0)	45 (2.4)	0.007
Hyperthyroidism	28 (1.7)	19 (1.1)	37 (2.3)	36 (1.9)	0.227
Risk factors for stroke					
Heart failure	370 (22.8)	487 (28.0)	468 (28.7)	569 (30.0)	<0.001
Hypertension	1008 (62.2)	1046 (60.1)	990 (60.7)	1132 (59.7)	0.200
Age (≥75 years)	525 (32.4)	633 (36.4)	560 (34.3)	669 (35.3)	0.210
Diabetes mellitus	255 (15.7)	342 (19.7)	296 (18.1)	372 (19.6)	0.016
Stroke/TIA	226 (13.9)	232 (13.3)	211 (12.9)	271 (14.3)	0.797
CHADS_2_ score	1.6 ± 1.2	1.7 ± 1.2	1.7 ± 1.2	1.7 ± 1.2	0.004
CHA_2_DS_2_-VASc score	2.7 ± 1.6	2.9 ± 1.6	2.8 ± 1.6	2.8 ± 1.6	0.086
HAS-BLED score (n = 6541)	1.5 ± 1.0	1.5 ± 1.0	1.5 ± 1.0	1.5 ± 1.0	0.179
Heart rate measurement times	14.5 ± 5.2	14.6 ± 5.2	14.7 ± 5.2	13.9 ± 5.2	0.001
Baseline heart rate, bpm	56.9 ± 4.4	67.0 ± 2.4	74.5 ± 2.5	89.2 ± 9.7	<0.001
Heart rate-end, bpm	66.9 ± 12.8	71.1 ± 11.3	74.6 ± 12.0	79.5 ± 13.6	<0.001
Systolic BP, mmHg	126.0 ± 16.1	125.5 ± 16.1	125.7 ± 15.6	126.7 ± 16.6	0.084
Diastolic BP, mmHg	71.4 ± 10.3	72.6 ± 11.1	74.5 ± 23.4	75.4 ± 11.3	<0.001
CrCl, mL/min (n = 5671)	68.6 ± 26.3	67.0 ± 27.0	69.1 ± 28.2	69.0 ± 29.1	0.515
Hemoglobin, g/dL (n = 6117)	13.6 ± 1.7	13.6 ± 1.8	13.8 ± 1.7	13.8 ± 1.8	<0.001
Medications					
Warfarin	1382 (85.3)	1450 (83.4)	1435 (88.0)	1664 (87.8)	0.001
PT-INR (n = 5931)	1.91 ± 0.50	1.90 ± 0.48	1.93 ± 0.52	1.90 ± 0.48	0.961
TTR*, % (n = 5611)	59.1 ± 29.3	60.4 ± 28.6	59.5 ± 29.4	58.6 ± 29.1	0.424
Antiplatelet	419 (25.8)	485 (27.9)	409 (25.1)	497 (26.2)	0.718
Aspirin	367 (22.6)	426 (24.5)	349 (21.4)	421 (22.2)	0.325
Warfarin + antiplatelet	287 (17.7)	330 (19.0)	293 (18.0)	348 (18.4)	0.829
ARB/ACE-I	872 (53.8)	913 (52.5)	879 (53.9)	999 (52.7)	0.725
Na channel blockers	312 (19.2)	368 (21.2)	331 (20.3)	402 (21.2)	0.288
β-blockers	250 (15.4)	286 (16.4)	248 (15.2)	296 (15.6)	0.799
K channel blockers**	226 (13.9)	271 (15.6)	233 (14.3)	255 (13.5)	0.374
Ca channel blockers	98 (6.0)	112 (6.4)	115 (7.1)	145 (7.7)	0.048
Digitalis	169 (10.4)	206 (11.8)	164 (10.1)	209 (11.0)	0.956

Data are number of patients (%) or mean ± SD.

bpm, beats per minute; HCM, hypertrophic cardiomyopathy; DCM, dilated cardiomyopathy; COPD, chronic obstructive pulmonary disease; TIA, transient ischemic attack; CHADS_2_, congestive heart failure, hypertension, age ≥ 75 years, diabetes mellitus, and history of stroke or TIA; CHA_2_DS_2_-VASc, additionally, vascular disease (coronary artery disease), age 65–74 years, and female sex; HAS-BLED, hypertension (systolic BP ≥ 140 mmHg), abnormal renal/liver function, stroke, bleeding history or predisposition, labile INR (episodes of INR ≥ 3.5), elderly (age > 65 years), drugs (use of antiplatelets)/alcohol concomitantly; heart rate-end, heart rate at the time closest to an event or at the last visit of follow-up; BP, blood pressure; CrCl, creatinine clearance; PT-INR, prothrombin time international normalized ratio; TTR, time in therapeutic range; ARB, angiotensin II receptor blocker; ACE-I, angiotensin converting enzyme inhibitor.

* Target PT-INR was 2.0–3.0 (<70 years) or 1.6–2.6 (≥70 years).

** Bepridil was classified as K channel blocker.

### 3.2 Event rates

During the two-year follow-up period, thromboembolism, major hemorrhage, all-cause death, and cardiovascular death occurred in 117 (1.7%), 130 (1.9%), 157 (2.3%), and 58 (0.8%) patients, respectively. The corresponding incidence rates of these events were 0.9, 0.9, 1.1, and 0.4/100 person-years, respectively, during a follow-up period of 13,758 person-years. Two-year event rates in the baseline HR quartiles, arbitrary HR groups, and HR-end quartiles are summarized in Table 2. None of the event rates in baseline HR (quartiles or arbitrary groups) showed significant trend across groups. In contrast, the rates of thromboembolism, major hemorrhage, and all-cause death showed significant trends across the HR-end quartiles (Table 2).

**Table 2:** Two-year event rates in HR groups.

**Baseline HR quartiles (bpm)**	**Lowest** **quartile** **(<63 bpm)**	**Second** **quartile** **(63–70 bpm)**	**Third** **quartile** **(71–79 bpm)**	**Highest** **quartile** **(≥80 bpm)**	***P*-value for trend**
Number of patients	1621	1739	1631	1895	
**Thromboembolism**	26 (1.6%)	33 (1.9%)	30 (1.8%)	28 (1.5%)	0.705
**Major hemorrhage**	27 (1.7%)	31 (1.8%)	34 (2.1%)	38 (2.0%)	0.376
**All-cause death**	34 (2.1%)	38 (2.2%)	33 (2.0%)	52 (2.7%)	0.241
**Cardiovascular death**	14 (0.9%)	17 (1.0%)	10 (0.6%)	17 (0.9%)	0.814


**Arbitrary baseline HR groups (bpm)**	**Lowest** **group** **(<60 bpm)**	**Second** **group** **(60–79 bpm)**	**Third** **group** **(80–109 bpm)**	**Highest** **group** **(≥110 bpm)**	***P*-value for trend**

Number of patients	884	4107	1802	93	
**Thromboembolism**	15 (1.7%)	74 (1.8%)	25 (1.4%)	3 (3.2%)	0.691
**Major hemorrhage**	13 (1.5%)	79 (1.9%)	37 (2.1%)	1 (1.1%)	0.480
**All-cause death**	19 (2.1%)	86 (2.1%)	48 (2.7%)	4 (4.3%)	0.140
**Cardiovascular death**	8 (0.9%)	33 (0.8%)	18 (0.9%)	1 (1.1%)	0.886


**HR-end quartiles (bpm)**	**Lowest** **quartile** **(<64 bpm)**	**Second** **quartile** **(64–71 bpm)**	**Third** **quartile** **(72–79 bpm)**	**Highest** **quartile** **(≥80 bpm)**	***P*-value for trend**

Number of patients	1583	1775	1575	1953	
**Thromboembolism**	15 (0.9%)	19 (1.1%)	34 (2.2%)	49 (2.5%)	<0.001
**Major hemorrhage**	23 (1.5%)	21 (1.2%)	16 (1.0%)	70 (3.6%)	<0.001
**All-cause death**	28 (1.8%)	24 (1.4%)	18 (1.1%)	87 (4.5%)	<0.001
**Cardiovascular death**	16 (1.0%)	5 (0.3%)	9 (0.6%)	28 (1.4%)	0.059

Data are number of patients (%).

HR, heart rate; bpm, beats per minute, HR-end, heart rate at the time closest to an event or at the last visit of follow-up.

### 3.3 Influence of HR on adverse events

**Fig. 1:**
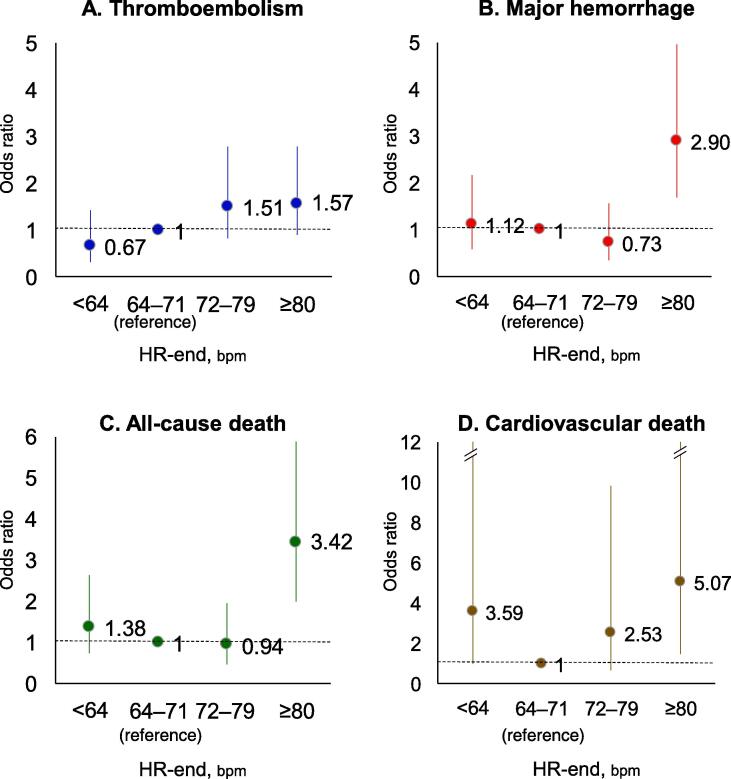
Odds ratios for thromboembolism (A), major hemorrhage (B), all-cause death (C), and cardiovascular death (D) in HR-end quartiles (multivariable analysis). HR-end, heart rate at the time closest to an event or at the last visit of follow-up Odds ratios were adjusted for components of CHA_2_DS_2_-VASc score, warfarin and antiplatelet use, type of atrial fibrillation, and blood pressure at the time closest to an event or at the last visit of follow-up, hypertrophic cardiomyopathy, chronic obstructive pulmonary disease, creatinine clearance, body mass index, hemoglobin level, and Ca channel blocker, β-blocker, and digitalis use.

In the univariable analysis, baseline HR, as a categorical or continuous variable, was not associated with any adverse event (Table 3). In contrast, for HR-end, ORs for thromboembolism in the third and highest quartiles, for major hemorrhage and all-cause death in the highest quartiles, and for cardiovascular death in the lowest and highest quartiles were significantly higher than those in the second quartile (Table 4). This was also true in the multivariable analysis with adjustment for components of CHA_2_DS_2_-VASc score, warfarin and antiplatelet use, AF type, and BP-end (Model 1) (Table 5). When the ORs were fully adjusted for confounding factors (Model 2), the ORs for major hemorrhage, all-cause death, and cardiovascular death in the highest quartile were significantly higher than those in the second quartile (Table 5 and Fig. 1). The OR for cardiovascular death in the lowest quartile remained marginally high (Table 5). When HR-end was analyzed as a continuous variable, the OR for HR-end (per 1-bpm increase) was significantly high for all events in univariable and multivariable analyses except for cardiovascular death in Model 2 (Table 4, Table 5).

**Table 3:** Hazard ratios for events in baseline HR quartiles (univariable analysis).

	**Thromboembolism**		**Major hemorrhage**		**All-cause death**		**Cardiovascular death**	
	**Hazard ratio** **(95% CI)**	***P*-value**	**Hazard ratio** **(95% CI)**	***P*-value**	**Hazard ratio** **(95% CI)**	***P*-value**	**Hazard ratio** **(95% CI)**	***P*-value**
**Lowest quartile** **(<63 bpm)**	0.85 (0.51–1.41)	0.522	0.94 (0.56–1.57)	0.800	0.96 (0.61–1.53)	0.868	0.89 (0.44–1.80)	0.735
**Second quartile** **(63–70 bpm)**	Reference	–	Reference	–	Reference	–	Reference	–
**Third quartile** **(71–79 bpm)**	0.96 (0.59–1.58)	0.885	1.17 (0.72–1.90)	0.537	0.92 (0.58–1.47)	0.734	0.63 (0.29–1.37)	0.238
**Highest quartile** **(≥80 bpm)**	0.79 (0.48–1.30)	0.351	1.14 (0.71–1.83)	0.598	1.27 (0.84–1.93)	0.261	0.93 (0.48–1.82)	0.831
**Baseline HR** **(/1-bpm increase)**	1.00 (0.99–1.01)	0.977	1.00 (0.96–1.01)	0.956	1.01 (1.00–1.02)	0.105	1.00 (0.98–1.02)	0.909

HR, hear rate; CI, confidence interval; bpm, beats per minute.

**Table 4:** Odds ratios for events in HR-end quartiles (univariable analysis).

	**Thromboembolism**		**Major hemorrhage**		**All-cause death**		**Cardiovascular death**	
	**OR** **(95% CI)**	***P*-value**	**OR** **(95% CI)**	***P*-value**	**OR** **(95% CI)**	***P*-value**	**OR** **(95% CI)**	***P*-value**
**Lowest quartile** **(<64 bpm)**	0.88 (0.45–1.75)	0.723	1.23 (0.68–2.23)	0.493	1.31 (0.46–2.28)	0.330	3.62 (1.32–9.89)	0.012
**Second quartile** **(64–71 bpm)**	Reference	–	Reference	–	Reference	–	Reference	–
**Third quartile** **(72–79 bpm)**	2.04 (1.16–3.59)	0.014	0.86 (0.45–1.35)	0.644	0.84 (0.46–1.56)	0.587	2.03 (0.68–6.08)	0.204
**Highest quartile** **(≥80 bpm)**	2.38 (1.40–4.06)	0.001	3.11 (1.90–5.08)	<0.001	3.40 (2.16–5.37)	<0.001	5.15 (1.98–13.36)	0.001
**HR-end** **(/1-bpm increase)**	1.03 (1.02–1.04)	<0.001	1.04 (1.03–1.05)	<0.001	1.04 (1.03–1.05)	<0.001	1.03 (1.01–1.04)	<0.001

HR, heart rate; HR-end, heart rate at the time closest to an event or at the last visit of follow-up; OR, odds ratio; CI, confidence interval; bpm, beats per minute.

**Table 5:** Odds ratios for events in HR-end quartiles (multivariable analysis).

	**Thromboembolism**		**Major hemorrhage**		**All-cause death**		**Cardiovascular death**	
	**OR** **(95% CI)**	***P*-value**	**OR** **(95% CI)**	***P*-value**	**OR** **(95% CI)**	***P*-value**	**OR** **(95% CI)**	***P*-value**
**Model 1**								
**Lowest quartile** **(<64 bpm)**	0.86 (0.43–1.71)	0.656	1.21 (0.66–2.20)	0.537	1.44 (0.81–2.57)	0.216	4.04 (1.46–11.21)	0.007
**Second quartile** **(64–71 bpm)**	Reference	–	Reference	–	Reference	–	Reference	–
**Third quartile** **(72–79 bpm)**	1.92 (1.08–3.41)	0.025	0.85 (0.44–1.64)	0.622	0.97 (0.51–1.83)	0.917	2.07 (0.69–6.25)	0.197
**Highest quartile** **(≥80 bpm)**	1.78 (1.03–3.08)	0.040	2.90 (1.76–4.77)	<0.001	3.58 (2.20–5.82)	<0.001	4.49 (1.70–11.81)	0.002
**HR-end** **(/1-bpm increase)**	1.02 (1.01–1.04)	<0.001	1.04 (1.03–1.05)	<0.001	1.04 (1.03–1.05)	<0.001	1.02 (1.00–1.03)	0.030
**Model 2**								
**Lowest quartile** **(<64 bpm)**	0.67 (0.32–1.43)	0.301	1.12 (0.58–2.16)	0.735	1.38 (0.73–2.63)	0.326	3.59 (0.98–13.22)	0.054
**Second quartile** **(64–71 bpm)**	Reference	–	Reference	–	Reference	–	Reference	–
**Third quartile** **(72–79 bpm)**	1.51 (0.83–2.78)	0.186	0.743 (0.35–1.56)	0.418	0.94 (0.46–1.95)	0.876	2.53 (0.65–9.83)	0.180
**Highest quartile** **(≥80 bpm)**	1.57 (0.89–2.79)	0.118	2.90 (1.69–4.96)	<0.001	3.42 (1.99–5.88)	<0.001	5.07 (1.49–17.22)	0.009
**HR-end** **(/1-bpm increase)**	1.03 (1.02–1.04)	<0.001	1.04 (1.03–1.05)	<0.001	1.03 (1.02–1.05)	<0.001	1.02 (1.00–1.04)	0.135

HR, heart rate; HR-end, heart rate at the time closest to an event or at the last visit of follow-up; OR, odds ratio; CI, confidence interval; bpm, beats per minute.

Model 1: adjusted for components of CHA_2_DS_2_-VASc score, warfarin and antiplatelet use, type of atrial fibrillation, and blood pressure at the time closest to an event or at the last visit of follow-up.

Model 2: adjusted for variables of Model 1 plus hypertrophic cardiomyopathy, chronic obstructive pulmonary disease, creatinine clearance, body mass index, hemoglobin level, and Ca channel blocker, β-blocker, and digitalis use (n = 5199).

### 3.4 Influence of HR on adverse events by AF type

When patients were divided into two subgroups based on baseline AF type, i.e., paroxysmal versus non-paroxysmal AF (Supplementary Table 1), all event rates in the baseline HR, either quartiles or arbitrary groups, showed no significant trend across groups, irrespective of AF type (Supplementary Tables 3 and 4). In contrast, the thromboembolism rate in patients with paroxysmal AF and the rates of major hemorrhage and all-cause death for both AF types showed significant trends across the HR-end quartiles (Supplementary Tables 3 and 4). The unadjusted ORs of the HR-end values for adverse events are shown in Supplementary Tables 5. After adjusting for the confounding factors in Model 2, the ORs for major hemorrhage in patients with paroxysmal AF and for major hemorrhage and all-cause and cardiovascular deaths in patients with non-paroxysmal AF were significantly higher in the highest HR-end quartile than in the second quartile (Supplementary Tables 6 and 7). When HR-end was analyzed as a continuous variable, the ORs for HR-end (per 1-bpm increase) were significantly higher for thromboembolism, major hemorrhage, and all-cause death in adjusted Model 2, irrespective of AF type (Supplementary Tables 6 and 7).

## 4 Discussion

The major findings of this study are as follows. First, baseline HR, as either a categorical or continuous variable, was not associated with any adverse event. Second, higher HR-end values were significantly associated with an increased incidence of adverse events. Third, the lowest quartile of HR-end was significantly associated with an increased incidence of cardiovascular death. Fourth, these findings remained almost consistent even when the patients were divided into paroxysmal and non-paroxysmal AF groups.

### 4.1 HR management in AF patients

HR management in patients with AF has been debated in terms of the superiority of rhythm control versus ventricular rate control strategies [15], [16] and the optimal target HR in the rate-control strategy [17], [18], [19]. In relation to the rhythm-control strategy, although previous studies demonstrated that the incidence of ischemic stroke appeared comparable between patients with paroxysmal and non-paroxysmal AF [20], [21], another study showed that the incidence of stroke or systemic embolism was lower in paroxysmal AF than in sustained AF [22]. In the recent Catheter Ablation versus Standard Conventional Therapy in Patients with Left Ventricular Dysfunction and Atrial Fibrillation (CASTLE-AF) trial [23], the incidence of a composite endpoint of all-cause death or hospitalization for worsening heart failure in patients who underwent catheter ablation was significantly lower than that in patients receiving medical therapy. On the other hand, in the rate-control strategy, HR has traditionally been controlled to the target of < 80 bpm at rest and < 110 (1 1 5) bpm during exercise. However, this was not proven in the RACE II trial [4]. Accordingly, current guidelines accept lenient HR control as the initial approach, and recommend adjusting the HR appropriately while considering subjective symptoms and QOL in each patient [5]. Thus, the impact of HR on adverse events in patients with NVAF remains controversial [4], [17], [18].

### 4.2 Impact of baseline HR on adverse events in NVAF patients

In the present study, first of all, we evaluated the impact of baseline HR on adverse events, since baseline HR was often analyzed in previous studies [17], [18], [19]. However, our results demonstrated that the baseline HR quartiles did not show a significant trend for any event across study groups (Table 2). In the Cox regression analysis, unadjusted hazard ratios of baseline HR evaluated as categorical or continuous variables showed no significant association with any adverse event (Table 3). Therefore, we abandoned to perform a multivariable analysis of baseline HR. In addition, even when the cutoff HR values of 80 and 110 bpm, the target HR values in the RACE II trial [4], were adopted for arbitrary baseline HR groups (<60, 60–79, 80–109, and ≥ 110 bpm), these groups also showed no significant trend for any event across the groups (Table 2). This may be explained by the fact that HR was controlled well at the time of enrollment, since stable outpatients with AF were enrolled in this registry. Indeed, 4107 patients (59.6%) were classified into the second group (HR 60–79 bpm), while only 93 (1.4%) patients were in the highest group (HR ≥ 110 bpm) (Table 2).

### 4.3 Impact of HR-end on adverse events in NVAF patients

Since we previously reported that baseline BP was not associated with the incidence of thromboembolism or major hemorrhage but systolic BP-end was significantly associated with these events in the same study cohort [9], HR-end was evaluated in this subanalysis. Consequently, the HR-end quartiles showed a significant trend for all adverse events across groups (Table 2). In the Cox regression analysis, unadjusted hazard ratios of the highest HR-end quartile and per 1-bpm increase in HR-end were significantly associated with an increased incidence of all adverse events (Table 4). Most remained significant even after adjusting for BP-end, indicating that the HR-end was significantly associated with adverse events independent of BP-end. In addition, these results were consistent even after adjusting for the use of CCB, which showed a significantly increasing trend across the baseline HR quartiles, and β-blockers and digitalis, which have a potential effect on HR. Interestingly, the hazard ratio of the lowest HR-end quartile was marginally associated with an increased incidence of cardiovascular death, even in the adjusted models (Table 5). This appeared to be a J-curve of the HR-end on cardiovascular death. Both tachycardia and bradycardia [17] could have affected cardiovascular death. The HR-end could represent the latest status of the changes in the balance between sympathetic and parasympathetic nerve activities, cardiac output, and hemodynamics during the follow-up period. Otherwise, acute tachycardiac or bradycardiac changes in HR at each visit would be a finding ominous of subsequent cardiovascular death. In any case, the HR-end value was more important and powerful than the baseline HR value for predicting adverse events in our cohort.

### 4.4 Influence of HR on adverse events and AF type

Since patients with paroxysmal AF had sinus rhythm during outpatient clinic visits, the influence of HR on adverse events might have differed from that in patients with non-paroxysmal AF. Thus, all analyses were performed separately for patients with paroxysmal and non-paroxysmal AF. As shown in Supplementary Table 1, the baseline HR, HR-end, and risk scores for thromboembolic and bleeding events were significantly higher in patients with non-paroxysmal versus paroxysmal AF. As the results from entire patients (Table 2), baseline HR was not associated with the incidence of any event, whereas HR-end was significantly associated with adverse events, irrespective of AF type (Supplementary Tables 3, 4, and 5), even in the adjusted models (Supplementary Tables 6 and 7). Overall, the impact of HR-end on adverse events was stronger in patients with non-paroxysmal versus paroxysmal AF.

### 4.5 Limitations

This study has several limitations. First, it was a *post hoc* analysis of the J-RHYTHM Registry [8], [24] and, therefore, hypothesis-generating in nature. Second, the study subjects were recruited from only 158 institutions in Japan, and most of the participating physicians specialized in cardiology and the management of cardiac arrhythmias. Therefore, these results may not be generalizable to the entire Japanese population with NVAF. In addition, because all study subjects were Japanese, the present results may not be applicable to other racial/ethnic groups. Third, changes in HR-controlling drugs and their doses, drug adherence, and progression from paroxysmal to persistent AF during the follow-up period were not considered in the present analyses. Direct oral anticoagulants were not approved for clinical use in Japan when the J-RHYHTM Registry was initiated. Therefore, the effects of direct oral anticoagulants on the relationship between HR and adverse events were not determined. Fourth, 1687 patients were excluded from Model 2 in the multivariable analysis due to missing data on CrCl, BMI, or hemoglobin levels. However, this exclusion might not have significantly influenced the results because there were no significant differences in two-year incidence rates of adverse events between patients included in and excluded from Model 2 (Supplementary Table 8). Fifth, the HR was not always recorded during AF, especially in patients with paroxysmal AF. This may have affected the results of entire patients. Therefore, all analyses were performed separately for patients with paroxysmal and non-paroxysmal AF to confirm the consistency with the results of entire cohort. Finally, although a significant association between HR-end and adverse events was found in this study, causality could not be determined. Moreover, the mechanisms of increased or decreased HR were not determined.

## 5 Conclusions

HR-end, but not baseline HR, was significantly associated with adverse events independent of systolic BP-end in patients with NVAF.

**Authors’ contribution**

Dr. Kodani contributed to the statistical analyses and wrote the manuscript. Drs. Inoue, Atarashi, Okumura, and Yamashita are on the Executive Committee of the J-RHYTHM Registry, and they planned and supervised the study comprehensively and edited the manuscript, as appropriate. Dr. Origasa contributed as a statistical advisor.

**Disclosures**

Dr. Kodani received remuneration from Daiichi-Sankyo and Ono Pharmaceutical; Dr. Inoue received remuneration from Boehringer Ingelheim, Bristol-Myers Squibb, and Daiichi-Sankyo; Dr. Atarashi received remuneration from Daiichi-Sankyo; Dr. Okumura received remuneration from Boehringer Ingelheim, Bristol-Myers Squibb, Daiichi-Sankyo, Johnson & Johnson, and Medtronic; and Dr. Yamashita received research funding from Bayer Healthcare, Bristol-Meyers Squibb, and Daiichi-Sankyo and remuneration from Bayer Healthcare, Bristol-Myers Squibb, Daiichi-Sankyo, Novartis, Ono Pharmaceutical, Otsuka Pharmaceutical, and Toa Eiyo.

## Declaration of Competing Interest

The authors declare that they have no known competing financial interests or personal relationships that could have appeared to influence the work reported in this paper.
