# Supplementary material for: Impact of heart rate on adverse events in patients with non-valvular atrial fibrillation: Subanalysis of the J-RHYTHM Registry
Source: Int J Cardiol Heart Vasc. 2022 Nov 17;43:101148. doi: 10.1016/j.ijcha.2022.101148 (PMC9678716; doi:10.1016/j.ijcha.2022.101148)
Supplement: Supplementary data 1 [file mmc1.pdf]

Supplementary Materials

Impact of heart rate on adverse events in patients with non-valvular atrial fibrillation: Subanalysis of the J-RHYTHM Registry

Eitaro Kodani, Hiroshi Inoue, Hirotsugu Atarashi, Ken Okumura, Takeshi Yamashita, Hideki Origasa, on behalf of the J-RHYTHM Registry Investigators

1. Supplementary Table 1. Patient characteristics and medications in each AF type
2. Supplementary Table 2. Patient characteristics and medications in HR-end quartiles
3. Supplementary Table 3. Two-year event rates in HR groups of paroxysmal AF
4. Supplementary Table 4. Two-year event rates in HR groups of non-paroxysmal AF
5. Supplementary Table 5. Odds ratios of HR-end for events in each AF type (univariable analysis)
6. Supplementary Table 6. Odds ratios of HR-end for events in paroxysmal AF (multivariable analysis)
7. Supplementary Table 7. Odds ratios of HR-end for events in non-paroxysmal AF (multivariable analysis)
8. Supplementary Table 8. Two-year event rates in patients included in and excluded from Model 2 of multivariable analysis

Supplementary Table 1. Patient characteristics and medications in each AF type

	Overall	Paroxysmal	Non-paroxysmal	*P*-value*
Number of patients	6886	2653	4233	
Age, years	69.8±9.9	68.4±10.3	70.6±9.7	<0.001
Sex, male	4874 (70.8)	1792 (67.5)	3082 (72.8)	<0.001
Body mass index, kg/m^2 (n=5979)	23.6±4.0	23.5±3.4	23.7±4.3	0.030
Type of atrial fibrillation				
Paroxysmal	2653 (38.5)	2653 (100)	0 (0)	
Persistent	1012 (14.7)	0 (0)	1012 (23.9)	<0.001
Permanent	3221 (46.8)	0 (0)	3221 (76.1)	
Comorbidities				
Coronary artery disease	724 (10.5)	268 (10.1)	456 (10.8)	0.377
Cardiomyopathy	573 (8.3)	154 (5.8)	419 (9.9)	<0.001
HCM	233 (3.4)	99 (3.7)	134 (3.2)	0.206
DCM	340 (4.9)	55 (2.1)	285 (6.7)	<0.001
Congenital heart disease	90 (1.3)	36 (1.4)	54 (1.3)	0.773
COPD	123 (1.8)	32 (1.2)	91 (2.1)	0.004
Hyperthyroidism120	120 (1.7)	34 (1.3)	86 (2.0)	0.021
Risk factors for stroke				
Heart failure	1794 (27.5)	380 (14.3)	1514 (35.8)	<0.001
Hypertension	4176 (60.6)	1600 (60.3)	2576 (60.9)	0.652
Age (≥75 years)	2387 (34.7)	808 (30.5)	1579 (37.3)	<0.001
Diabetes mellitus	1265 (18.4)	386 (14.5)	879 (20.8)	<0.001
Stroke/TIA	940 (13.7)	307 (11.6)	633 (15.0)	<0.001
$CHADS_2$ score	1.7±1.2	1.4±1.1	1.9±1.3	<0.001
CHA_2DS_2-VASc score	2.8±1.6	2.5±1.5	3.0±1.6	<0.001
HAS-BLED score (n=6541)	1.5±1.0	1.4±1.0	1.6±1.0	<0.001
Heart rate measurement times	14.4±5.2	14.2±5.1	14.5±5.2	0.014
Baseline heart rate, bpm	72.5±13.3	68.5±12.7	75.0±13.0	<0.001
Heart rate-end, bpm	73.3±13.3	70.6±12.9	75.0±13.3	<0.001
Systolic BP, mmHg	126.0±16.1	127.2±16.1	125.2±16.1	<0.001
Diastolic BP, mmHg	73.5±14.9	73.3±19.1	73.6±11.5	0.405
CrCl, mL/min (n=5671)	68.4±27.7	70.8±28.4	67.0±27.2	<0.001
Hemoglobin, g/dL (n=6117)	13.7±1.7	13.5±1.7	13.8±1.8	<0.001
Medications				
Warfarin	5931 (86.1)	2072 (78.1)	3859 (91.2)	<0.001
PT-INR (n=5931)	1.91±0.49	1.88±0.49	1.92±0.50	0.004
TTR**, % (n=5611)	59.4±29.1	57.5±29.5	60.4±28.8	<0.001
Antiplatelet	1810 (26.3)	646 (24.3)	1164 (27.5)	0.004
Aspirin	1653 (22.7)	591 (22.3)	972 (23.0)	0.509
Warfarin+antiplatelet	1258 (18.3)	374 (14.1)	884 (20.9)	<0.001
ARB/ACE-I	3663 (53.2)	1306 (49.2)	2357 (55.7)	<0.001
Na channel blockers	1413 (20.5)	547 (20.6)	866 (20.5)	0.823
β-blockers	1080 (15.7)	403 (15.2)	677 (16.0)	0.399
K channel blockers***	985 (14.3)	367 (13.8)	618 (14.6)	0.402
Ca channel blockers	470 (6.8)	175 (6.6)	295 (7.0)	0.572
Digitalis	748 (10.9)	302 (11.4)	446 (10.5)	0.252

Data are number of patients (%) or mean±SD. Abbreviations are as in Table 1.

* Comparison between paroxysmal and non-paroxysmal.

** Target PT-INR was 2.0–3.0 (<70 years) or 1.6–2.6 (≥70 years).

*** Bepridil was classified as K channel blocker.

Supplementary Table 2. Patient characteristics and medications in HR-end quartiles

	Lowest (<64 bpm)	Second (64–71 bpm)	Third (72–79 bpm)	Highest (≥80 bpm)	*P*-value for trend
Number of patients	1583	1775	1575	1953	
Age, years	69.6±9.5	69.7±9.6	69.7±10.2	70.0±10.4	0.027
Sex, male	1156 (73.0)	1257 (70.8)	1108 (70.3)	1353 (69.3)	0.018
Body mass index, kg/m^2 (n=5979)	23.6±3.4	23.7±3.7	23.6±3.4	23.6±5.0	0.475
Type of atrial fibrillation					
Paroxysmal	816 (51.5)	759 (42.8)	532 (33.8)	546 (28.0)	
Persistent	214 (13.5)	245 (13.8)	234 (14.9)	319 (16.3)	<0.001
Permanent	553 (34.9)	771 (43.4)	809 (51.4)	1088 (55.7)	
Comorbidities					
Coronary artery disease	173 (10.9)	198 (11.2)	169 (10.7)	184 (9.4)	0.110
Cardiomyopathy	127 (8.0)	155 (8.7)	123 (7.8)	168 (8.6)	0.768
HCM	65 (4.1)	72 (4.1)	44 (2.8)	52 (2.7)	0.004
DCM	62 (3.9)	83 (4.7)	79 (5.0)	116 (5.9)	0.005
Congenital heart disease	25 (1.6)	19 (1.1)	27 (1.7)	19 (1.0)	0.303
COPD	24 (1.5)	30 (1.7)	24 (1.5)	45 (2.3)	0.100
Hyperthyroidism	26 (1.6)	39 (2.2)	27 (1.7)	28 (1.4)	0.372
Risk factors for stroke					
Heart failure	377 (23.8)	473 (26.6)	435 (27.6)	609 (31.2)	<0.001
Hypertension	969 (61.2)	1043 (58.8)	956 (60.7)	120.8 (61.9)	0.393
Age (≥75 years)	511 (32.3)	602 (33.9)	540 (34.3)	734 (37.6)	0.001
Diabetes mellitus	273 (17.2)	305 (17.2)	299 (19.0)	388 (19.9)	0.018
Stroke/TIA	216 (13.6)	261 (14.7)	189 (12.0)	274 (14.0)	0.730
$CHADS_2$ score	1.6±1.2	1.7±1.3	1.7±1.2	1.8±1.3	<0.001
CHA_2DS_2-VASc score	2.7±1.6	2.8±1.6	2.8±1.5	2.9±1.6	<0.001
HAS-BLED score (n=6541)	1.5±1.0	1.5±1.0	1.4±1.0	1.6±1.0	0.005
Heart rate measurement times	14.8±5.1	14.5±5.2	14.6±5.1	13.8±5.2	<0.001
Baseline heart rate, bpm	65.5±11.8	70.5±11.2	74.1±11.8	78.8±14.1	<0.001
Heart rate-end, bpm	57.8±4.4	67.7±2.2	74.8±2.4	89.6±10.4	<0.001
Systolic BP, mmHg	126.8±16.6	125.2±15.4	125.9±15.6	126.0±16.8	0.494
Diastolic BP, mmHg	72.7±10.6	73.2±22.3	74.1±10.7	74.0±12.6	<0.001
CrCl, mL/min (n=5671)	69.0±26.0	69.0±26.5	68.7±28.0	67.2±29.8	0.004
Hemoglobin, g/dL (n=6117)	13.6±1.6	13.7±1.7	13.8±1.7	13.7±1.8	0.041
Medications					
Warfarin	1361 (86.0)	1501 (84.6)	1381 (87.7)	1688 (86.4)	0.233
PT-INR (n=5931)	1.90±0.50	1.92±0.50	1.91±0.51	1.90±0.47	0.683
TTR*, % (n=5611)	59.0±28.9	61.0±28.9	59.3±29.2	58.3±29.3	0.234
Antiplatelet	389 (24.6)	479 (27.0)	379 (24.1)	563 (28.8)	0.027
Aspirin	337 (21.3)	436 (24.6)	312 (19.8)	478 (24.5)	0.239
Warfarin+antiplatelet	268 (16.9)	332 (18.7)	273 (17.3)	385 (19.7)	0.082
ARB/ACE-I	858 (54.2)	918 (51.7)	840 (53.3)	1047 (53.6)	0.958
Na channel blockers	316 (21.0)	379 (22.5)	302 (20.3)	416 (22.3)	0.654
β-blockers	248 (16.5)	270 (16.0)	261 (17.5)	301 (16.2)	0.937
K channel blockers**	210 (14.0)	288 (17.1)	232 (15.6)	255 (13.7)	0.418
Ca channel blockers	92 (6.1)	114 (6.8)	114 (7.7)	150 (8.1)	0.019
Digitalis	177 (11.8)	185 (11.0)	164 (11.0)	222 (11.9)	0.812

Data are number of patients (%) or mean±SD. Abbreviations are as in Table 1.

* Target PT-INR was 2.0–3.0 (<70 years) or 1.6–2.6 (≥70 years).

** Bepridil was classified as K channel blocker.

Supplementary Table 3. Two-year event rates in HR groups of paroxysmal AF

Baseline HR quartiles (bpm)	Lowest quartile (<60 bpm)	Second quartile (60–66 bpm)	Third quartile (67–73 bpm)	Highest quartile (≥74 bpm)	*P*-value for trend
Number of patients	515	786	648	704	
**Thromboembolism**	7 (1.4%)	6 (0.8%)	5 (0.8%)	10 (1.4%)	0.760
**Major hemorrhage**	7 (1.4%)	12 (1.5%)	8 (1.2%)	12 (1.7%)	0.726
**All-cause death**	6 (1.2%)	9 (1.1%)	15 (2.3%)	11 (1.6%)	0.294
**Cardiovascular death**	2 (0.4%)	3 (0.4%)	5 (0.8%)	3 (0.4%)	0.709
**Arbitrary baseline HR groups (bpm)**	**Lowest group (<60 bpm)**	**Second group (60–79 bpm)**	**Third group (80–109 bpm)**	**Highest group (≥110 bpm)**	***P*-value for trend**
Number of patients	515	1690	422	26	
**Thromboembolism**	7 (1.4%)	15 (0.9%)	6 (1.4%)	0 (0.0%)	0.863
**Major hemorrhage**	7 (1.4%)	26 (1.5%)	5 (1.2%)	1 (3.8%)	0.877
**All-cause death**	6 (1.2%)	29 (1.7%)	6 (1.4%)	0 (0.0%)	0.873
**Cardiovascular death**	2 (0.4%)	9 (0.5%)	2 (0.5%)	0 (0.0%)	0.929
**HR-end quartiles (bpm)**	**Lowest quartile (<66 bpm)**	**Second quartile (66–72 bpm)**	**Third quartile (73–81 bpm)**	**Highest quartile (≥82 bpm)**	***P*-value for trend**
Number of patients	648	626	690	689	
**Thromboembolism**	5 (0.8%)	4 (0.6%)	8 (1.2%)	11 (1.6)	0.090
**Major hemorrhage**	4 (0.6%)	6 (1.0%)	3 (0.4%)	26 (3.8%)	<0.001
**All-cause death**	6 (0.9%)	4 (0.6%)	9 (1.3%)	22 (3.2%)	<0.001
**Cardiovascular death**	3 (0.5%)	1 (0.2%)	2 (0.3%)	7 (1.0%)	0.134

Data are number of patients (%).
HR, heart rate; bpm, AF, atrial fibrillation; beats per minute, HR-end, heart rate at the time closest to an event or at the last visit of follow-up.

Supplementary Table 4. Two-year event rates in HR groups of non-paroxysmal AF

**Baseline HR quartiles (bpm)**	**Lowest quartile (<66 bpm)**	**Second quartile (66–72 bpm)**	**Third quartile (73–82 bpm)**	**Highest quartile (≥83 bpm)**	***P*-value for trend**
Number of patients	948	1099	1111	1075	
**Thromboembolism**	21 (2.2%)	25 (2.3%)	25 (2.3%)	18 (1.7%)	0.399
**Major hemorrhage**	21 (2.2%)	20 (1.8%)	31 (2.8%)	19 (1.8%)	0.868
**All-cause death**	26 (2.7%)	29 (2.6%)	25 (2.3%)	36 (3.3%)	0.513
**Cardiovascular death**	11 (1.2%)	14 (1.3%)	8 (0.7%)	12 (1.1%)	0.624
**Arbitrary baseline HR groups (bpm)**	**Lowest group (<60 bpm)**	**Second group (60–79 bpm)**	**Third group (80–109 bpm)**	**Highest group (≥110 bpm)**	***P*-value for trend**
Number of patients	369	2417	1380	67	
**Thromboembolism**	8 (2.2%)	59 (2.4%)	19 (1.4%)	3 (4.5%)	0.233
**Major hemorrhage**	5 (1.6%)	53 (2.2%)	32 (2.3%)	0 (0.0%)	0.817
**All-cause death**	13 (3.5%)	57 (2.4%)	42 (3.0%)	4 (6.0%)	0.405
**Cardiovascular death**	6 (1.6%)	24 (1.0%)	14 (1.0%)	1 (1.5%)	0.608
**HR-end quartiles (bpm)**	**Lowest quartile (<66 bpm)**	**Second quartile (66–72 bpm)**	**Third quartile (73–81 bpm)**	**Highest quartile (≥82 bpm)**	***P*-value for trend**
Number of patients	942	1172	970	1149	
**Thromboembolism**	10 (1.1%)	20 (1.7%)	23 (2.3%)	36 (3.1%)	0.001
**Major hemorrhage**	21 (2.2%)	12 (1.0%)	13 (1.3%)	45 (3.9%)	0.002
**All-cause death**	26 (2.8%)	16 (1.4%)	17 (1.8%)	57 (5.0%)	<0.001
**Cardiovascular death**	14 (1.5%)	5 (0.4%)	10 (1.0%)	16 (1.4%)	0.659

Data are number of patients (%).
HR, heart rate; bpm, AF, atrial fibrillation; beats per minutes, HR-end, heart rate at the time closest to an event or at the last visit of follow-up.

Supplementary Table 5. Odds ratios of HR-end for events in each AF type (univariable analysis)

	Thromboembolism		Major hemorrhage		All-cause death		Cardiovascular death	
	**OR (95% CI)**	***P*-value**	**OR (95% CI)**	***P*-value**	**OR (95% CI)**	***P*-value**	**OR (95% CI)**	***P*-value**
**Paroxysmal AF**								
**Lowest quartile (<61 bpm)**	1.21 (0.32–4.52)	0.778	0.64 (0.18–2.29)	0.494	1.45 (0.41–5.18)	0.564	2.91 (0.30–28.02)	0.356
**Second quartile (61–68 bpm)**	Reference	-	Reference	-	Reference	-	Reference	-
**Third quartile (69–76 bpm)**	1.82 (0.55–6.09)	0.328	0.45 (0.11–1.81)	0.292	2.06 (0.63–6.71)	0.233	1.82 (0.16–20.09)	0.626
**Highest quartile (≥77 bpm)**	2.52 (0.80–7.96)	0.115	4.05 (1.66–9.91)	0.002	5.13 (1.76–14.97)	0.003	6.42 (0.79–52.29)	0.083
**HR-end (/1-bpm increase)**	1.04 (1.01–1.06)	0.001	1.05 (1.03–1.06)	<0.001	1.06 (1.04–1.07)	<0.001	1.06 (1.03–1.09)	<0.001
**Non-paroxysmal AF**								
**Lowest quartile (<66 bpm)**	0.62 (0.29–1.33)	0.217	2.20 (1.08–4.50)	0.030	2.05 (1.09–3.85)	0.025	3.52 (1.26–9.81)	0.016
**Second quartile (66–72 bpm)**	Reference	-	Reference	-	Reference	-	Reference	-
**Third quartile (73–81 bpm)**	1.40 (0.76–2.56)	0.431	1.31 (0.60–2.89)	0.499	1.29 (0.65–2.57)	0.470	2.43 (0.83–7.14)	0.106
**Highest quartile (≥82 bpm)**	1.86 (1.07–3.24)	0.027	3.94 (2.07–7.49)	<0.001	3.77 (2.15–6.61)	<0.001	3.30 (1.20–9.03)	0.020
**HR-end (/1-bpm increase)**	1.03 (1.01–1.04)	<0.001	1.03 (1.02–1.04)	<0.001	1.04 (1.03–1.05)	<0.001	1.01 (0.99–1.03)	0.336

HR, heart rate; HR-end, heart rate at the time closest to an event or at the last visit of follow-up; AF, atrial fibrillation; OR, odds ratio; CI, confidence interval; bpm, beats per minute.

Supplementary Table 6. Odds ratios of HR-end for events in paroxysmal AF (multivariable analysis)

	Thromboembolism		Major hemorrhage		All-cause death		Cardiovascular death	
	OR (95% CI)	*P*-value	OR (95% CI)	*P*-value	OR (95% CI)	*P*-value e	OR (95% CI)	*P*-value
**Multivariable (Model 1)**								
**Lowest quartile (<61 bpm)**	1.18 (0.31–4.51)	0.813	0.65 (0.18–2.33)	0.507	1.36 (0.37–4.94)	0.645	3.23 (0.32–32.35)	0.356
**Second quartile (61–68 bpm)**	Reference	-	Reference	-	Reference	-	Reference	-
**Third quartile (69–76 bpm)**	1.92 (0.56–6.53)	0.299	0.47 (0.12–1.88)	0.283	1.58 (0.47–5.33)	0.466	1.94 (0.17–22.02)	0.606
**Highest quartile (≥77 bpm)**	2.22 (0.69–7.16)	0.183	4.12 (1.66–10.24)	0.002	4.10 (1.35–12.45)	0.013	5.12 (0.59–44.52)	0.139
**HR-end (/1-bpm increase)**	1.03 (1.01–1.06)	0.010	1.05 (1.03–1.06)	<0.001	1.05 (1.03–1.07)	<0.001	1.05 (1.02–1.08)	0.003
**Multivariable (Model 2)**								
**Lowest quartile (<61 bpm)**	0.95 (0.23–3.87)	0.940	0.37 (0.10–1.46)	0.157	0.94 (0.28–3.13)	0.913	1.58 (0.13–19.90)	0.723
**Second quartile (61–68 bpm)**	Reference	-	Reference	-	Reference	-	Reference	-
**Third quartile (69–76 bpm)**	2.23 (0.61–8.14)	0.224	1.20 (0.40–3.61)	0.750	0.87 (0.24–3.22)	0.837	3.45 (0.27–44.06)	0.340
**Highest quartile (≥77 bpm)**	2.25 (0.65–7.83)	0.202	3.15 (1.27–7.80)	0.013	2.20 (0.76–6.33)	0.145	3.56 (0.34–37.58)	0.291
**HR-end (/1-bpm increase)**	1.04 (1.01–1.06)	0.007	1.05 (1.03–1.07)	<0.001	1.03 (1.01–1.06)	0.012	1.03 (0.98–1.08)	0.222

HR, heart rate; HR-end, heart rate at the time closest to an event or at the last visit of follow-up; AF, atrial fibrillation; OR, odds ratio; CI, confidence interval; bpm, beats per minute.

Model 1: adjusted for components of CHA_2DS_2-VASc score, warfarin and antiplatelet use, type of atrial fibrillation, and blood pressure at the time closest to an event or at the last visit of follow-up.

Model 2: adjusted for variables of Model 1 plus hypertrophic cardiomyopathy, chronic obstructive pulmonary disease, creatinine clearance, body mass index, hemoglobin level, and Ca channel blocker, β-blocker, and digitalis use (n=1983).

Supplementary Table 7. Odds ratios of HR-end for events in non-paroxysmal AF (multivariable analysis)

	Thromboembolism		Major hemorrhage		All-cause death		Cardiovascular death	
	**OR (95% CI)**	***P*-value**	**OR (95% CI)**	***P*-value**	**OR (95% CI)**	***P*-value**	**OR (95% CI)**	***P*-value**
**Multivariable (Model 1)**								
**Lowest quartile (<66 bpm)**	0.57 (0.26–1.25)	0.160	2.12 (1.03–4.35)	0.041	2.26 (1.16–4.41)	0.017	3.74 (1.32–10.57)	0.013
**Second quartile (66–72 bpm)**	Reference	-	Reference	-	Reference	-	Reference	-
**Third quartile (73–81 bpm)**	1.28 (0.69–2.37)	0.431	1.34 (0.61–2.97)	0.467	1.65 (0.80–3.40)	0.175	2.65 (0.89–7.88)	0.081
**Highest quartile (≥82 bpm)**	1.45 (0.82–2.57)	0.198	3.90 (2.04–7.46)	<0.001	4.55 (2.49–8.33)	<0.001	3.09 (1.10–8.67)	0.032
**HR-end (/1-bpm increase)**	1.02 (1.01–1.04)	0.005	1.03 (1.02–1.05)	<0.001	1.04 (1.02–1.05)	<0.001	1.01 (0.99–1.03)	0.541
**Multivariable (Model 2)**								
**Lowest quartile (<66 bpm)**	0.77 (0.33–1.81)	0.555	1.42 (0.65–3.08)	0.381	1.64 (0.79–3.41)	0.182	2.36 (0.70–7.95)	0.166
**Second quartile (66–72 bpm)**	Reference	-	Reference	-	Reference	-	Reference	-
**Third quartile (73–81 bpm)**	1.24 (0.62–2.51)	0.545	0.54 (0.20–1.43)	0.214	0.86 (0.37–2.02)	0.729	1.40 (0.37–5.24)	0.621
**Highest quartile (≥82 bpm)**	1.50 (0.80–2.83)	0.209	2.47 (1.29–4.74)	0.010	3.69 (2.00–6.79)	<0.001	3.16 (1.05–9.49)	0.040
**HR-end (/1-bpm increase)**	1.03 (1.01–1.04)	0.001	1.03 (1.02–1.05)	<0.001	1.03 (1.02–1.04)	<0.001	1.01 (0.99–1.04)	0.249

HR, heart rate; HR-end, heart rate at the time closest to an event or at the last visit of follow-up; AF, atrial fibrillation; OR, odds ratio; CI, confidence interval; bpm, beats per minute.

Model 1: adjusted for components of CHA_2DS_2-VASc score, warfarin and antiplatelet use, type of atrial fibrillation, and blood pressure at the time closest to an event or at the last visit of follow-up.

Model 2: adjusted for variables of Model 1 plus hypertrophic cardiomyopathy, chronic obstructive pulmonary disease, creatinine clearance, body mass index, hemoglobin level, and Ca channel blocker, β-blocker, and digitalis use (n=3407).

Supplementary Table 8. Two-year event rates in patients included in and excluded from Model 2 of multivariable analysis

	Overall	Included in Model 2 (Including CrCl, BMI, and Hb)	Excluded from Model 2 (Missing CrCl, BMI, or Hb)	*P*-value*
Number of patients	6886	5199	1687	
**Thromboembolism**	117 (1.7%)	95 (1.8%)	22 (1.3%)	0.149
**Major hemorrhage**	130 (1.9%)	103 (2.0%)	27 (1.6%)	0.318
**All-cause death**	157 (2.3%)	118 (2.3%)	39 (2.3%)	0.920
**Cardiovascular death**	58 (0.8%)	40 (0.8%)	18 (1.1%)	0.245

* Comparison between included in and excluded from Model 2.

Data are number of patients (%).

CrCl, creatinine clearance; BMI, body mass index; Hb, hemoglobin.
